# Production, Optimization, and Characterization of an Acid Protease from a Filamentous Fungus by Solid-State Fermentation

**DOI:** 10.1155/2021/6685963

**Published:** 2021-04-29

**Authors:** Abdilbar Usman, Said Mohammed, Jermen Mamo

**Affiliations:** Department of Biology, College of Natural and Computational Science, Debre Berhan University, Debre Berhan, Ethiopia

## Abstract

Acid proteases represent an important group of enzymes, extensively used in food and beverage industries. There is an increased demand for acid proteases adapting to the industrial extreme environment, especially lower pH. Thus, this necessitates the search for a better acid protease from fungi that best performs in industrial conditions. The fungal isolates were isolated from grape and dairy farm soil using potato dextrose agar and further screened for protease production based on the hydrolysis of clear zone on skim milk agar. The potential fungi were then subjected to secondary screening under solid-state fermentation (SSF). After the secondary screening, the potential fungus was identified to the genus level by the macroscopic and microscopic methods. The growth conditions and media composition for the potential fungus were further optimized under SSF. The crude enzyme produced by the potential isolate was characterized after partial purification by acetone and ammonium sulfate precipitation. A total of 9 fungal isolates showed protease production in primary and secondary screening; however, one potential isolate (Z1BL1) was selected for further study based on its protease activity. The isolate was identified to the genus *Aspergillus* based on their morphological features. The maximum acid protease from the isolate Z1BL1 was obtained using fermentation media containing wheat bran as a solid substrate, 1 mL of 3.2 *×* 10^6^ inoculum size, 50% moisture content, and pH 4.5 upon 120-h incubation at 30°C. The acetone-precipitated enzyme exhibited the maximum activity at 50°C and pH 5 with stability at pH 4–6 and temperature 40–60°C. Thus, the acid protease produced from *Aspergillus* showed suitable enzyme characteristics required in the industry and could be a candidate for application in the food industry after further purification.

## 1. Introduction

Proteases are the most essential group of enzymes from biotechnological perspectives, and they catalyze the hydrolysis of peptide bonds [1, 2]. Proteases account for about 65% of the total worldwide enzyme sales, and they are made up of a complex group of enzymes and differ in some of their properties such as substrate specificity, catalytic mechanism, temperature, and pH optima and stability profile [2].

Aspartic proteases (EC 3.4.23), also known as acidic proteases, are a subfamily of endopeptidases that have been isolated from diverse sources, including viruses, bacteria, fungi, plants, and animals [1]. Several fungal aspartic proteases have been purified and characterized as rennin-like and pepsin-like enzymes [3]. The rennin-like enzymes are produced by *Endothia parasitica* (endothiapepsin, EC 3.4.23.22), *Mucor*, and *Rhizomucor* species (mucorpepsin, EC 3.4.23.23). The pepsin-like enzymes include aspergillopepsin (aspergillopepsin I, EC 3.4.23.18) from *Aspergillus* species [4] and rhizopuspepsin (EC 3.4.23.21) from *Rhizopus* species [1].

Most of the aspartic proteases (Aps) show the best activity at low pH (pH 3 to 4) and have isoelectric points in the pH range of 3 to 4.5 [5]. They are inhibited by a hexapeptide from *Streptomyces* that contains two statin residues called pepstatin. Aspartic proteases are also sensitive to diazoacetyl-DL-norleucine methyl ester (DAN) and 1, 2-epoxy-3-(*p*-nitrophenoxy) propane (EPNP) in the presence of copper ions. Microbial acid proteases exhibit specificity against aromatic or bulky amino acid residues on both sides of the peptide bond, which is similar to pepsin, but their action is less stringent than that of pepsin [5].

Acid proteases have a wide range of applications in various industries such as food industry, beverage industry, and pharmaceutical industry [5]. The increasing importance of these enzymes and their numerous applications in different industries improves the effort to investigate acid protease with enhanced activity from fungi. The shortage of plant and animal proteases to meet the present world demands of industrial enzymes has directed an increased interest in microbial proteases [5, 6]. The diverse ecological niche in Ethiopia and the presence of very few reports on fungal acid proteases from Ethiopia [7] necessitate the search for potential fungal isolates with improved acid protease activity. Therefore, the present study was intended to isolate and screen fungi from soil samples for the production, optimization, and characterization of acid protease.

## 2. Materials and Methods

### 2.1. Description of Study Area

A total of 21 soil samples were collected from dairy and grape farms in three regional states of Ethiopia, i.e, Amhara Region (Shewa Robit); Oromia Region (Ziway); and Southern Nations, Nationalities, People's Region (Halaba). Shewa Robit is located at 100 06′ 650″–090 57′957″ N, 0390 54′37″–0390 56′579″ E, in North Shewa Zone of the Amhara regional state, 225 km north of Addis Ababa, Ethiopia. It is a low-land (submoist warm) area with altitudes ranging between 1120 and 1350 m above sea level. The climate data of the study area recorded for the last ten years show an average annual maximum and minimum temperature and precipitation of 32.1 and 16.1°C and 968 mm, respectively. The most dominant soil type in Shewa Robit is vertisols, which have clay texture with a clay content of 56% and 10%, silt 34%, and a pH value of 8.02 [8].

The second study area Ziway is located at 7.58°N latitude and 38.43°E longitude in the southern part of the Oromia regional state situated in the Mid Rift Valley, 160 km south of Addis Ababa. It is categorized under the semiarid, with a minimum mean temperature of 12.7°C and a maximum mean temperature of 27.2°C with a relative humidity of 60%. The area has an altitude ranging between 1500 and 2000 meters above sea level. The average annual rainfall ranges from 650 to 750 mm, and the distribution is highly variable between and within years. Vertisol is the predominant soil type with sand-silt-clay in the portion of 33 : 48 : 18, respectively, and has a pH of 7.88 [9].

**Figure 1:**
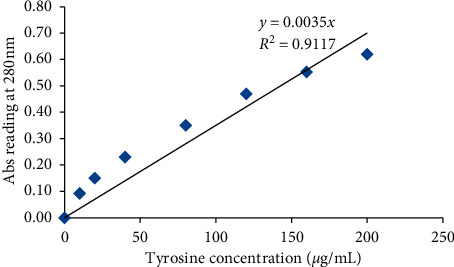
Tyrosine standard curve for acid protease activity.

Halaba special district is located in Southern Nation, Nationalities and Peoples Region (SNNPR), at the distance of 85 km from Hawassa town and 310 km from Addis Ababa, the capital city of Ethiopia (Figure 1). The study site is found within an altitude ranging from 1554 to 2149 m above sea level, and an astronomic location of 38° 7′0″E longitude and 7°18′0″N latitude. Halaba special district is generally characterized by dry climatic condition with about 86% mid-land (Weinadega) and 14% law-land (Kola) zones. The mean annual rainfall of the study area is ranging from 857 to 1085 mm, while the mean annual temperature varies from 17 to 20°C with a mean value of 18°C [10].

### 2.2. Sample Collection

Five hundred grams of soil (5–10 cm below the surface) was collected aseptically from the grape farm and dairy farm areas located at Showa Robit, Ziway, and Halaba, Ethiopia, in September 2019. Soil samples were sieved (3–4 mm mesh), homogenized, and stored at 4°C at the Microbiology Laboratory of the Biology Department, Debre Berhan University, for further use as the protocol described by Li et al. [11].

### 2.3. Isolation of Fungi

The fungi were isolated by serial dilution technique as described by Oyeleke et al. [12]. Thus, ten grams of each soil sample was mixed with 90 mL of distilled water and homogenized by agitation for 20 min. They were prepared to appropriate dilutions from which 0.1 mL of each sample suspension was plated on potato dextrose agar (PDA) (Microgen, India) plates containing (0.05 g/L) chloramphenicol. The plates were incubated at 30°C for 5–7 days to isolate distinctive colonies. Each colony was then restreaked on the same medium to purity and preserved at 4°C.

### 2.4. Primary Screening for Protease-Producing Fungi

Primary screening for protease production was tested using the skim milk agar (Nestle TM, Frankfurt, Germany) medium for the production of the clear zone [13]. The detection medium (Skim Milk Agar, SMA) was prepared using 20 g of skim milk, 20 g of agar-agar each dissolved in 200 mL distilled water, and 600 mL of 0.2 M phosphate buffer (K_2_HPO_4_ and KH_2_PO_4_, pH 5.0). All the three media components were autoclaved separately to avoid coagulation and charring of milk due to the presence of buffer salts and later mixed under sterile conditions. The plates were then subsequently inoculated with mycelia from 5-day-old culture and incubated at 30°C for 2 days. The plates were examined for the formation of the clearing zone by flooding them with a solution of 10% trichloroacetic acid (TCA) or 10% tannic acid. The relative enzyme activity was calculated using the following formula:

(1) $$\begin{array}{c} \text{REA}=\frac{\text{CZD}}{\text{CD}}, \end{array}$$

where REA: relative enzyme activity, CZD: clear zone diameter, and CD: colony diameter [13, 14].

### 2.5. Secondary Screening for Acid Protease under Solid-State Fermentation

#### 2.5.1. Inoculum Preparation

Fungal isolates were grown on PDA and incubated at 30°C for 5 days. They were scrapped using 10 mL of sterile distilled water to prepare spore suspension. 0.5 mL of spore solution (10^6^ spores/mL) was used according to the study by Sathya et al. [15].

#### 2.5.2. Medium and Cultural Conditions for Solid-State Fermentation for Fungi (M1)

For solid-state fermentation, 0.5 mL of spore suspension (10^6^ spores/mL) was transferred into 250 mL Erlenmeyer flasks containing wheat bran (10 g), skim milk powder (2.0 g), and 10 mL of salt solution (g/L: 2.0, KNO_3_; 0.5, MgSO_4_·7H_2_O; 1.0, K2HPO_4_; 0.439, ZnSO_4_·7H_2_O; 1.116, FeSO_4_·7H_2_O; 0.203, MnSO_4_·7H_2_O, and pH 5) [15]. The flasks were incubated at 30°C for 6 days under static conditions.

#### 2.5.3. Medium and Cultural Conditions for Solid-State Fermentation for Fungi (M2)

The solid-state substrate was also prepared in 250 mL Erlenmeyer flasks containing 10 g of wheat bran (Durum wheat bran) moistened by adding 12 mL of HCl (0.2 M) and mixed thoroughly. Then, the flasks were autoclaved at 121°C for 30 min. Then, 0.5 mL of (10^6^ spores/mL) spore suspension was inoculated into SSF media and incubated at 30°C for 6 days [16].

### 2.6. Enzyme Extraction

The enzyme was extracted according to the method described by Silveira et al. [17]. Thus, the fermented substrates were dispersed in 100 mL of distilled water (1 : 10 ratio of Bran-solvent w/v) and vigorously shaken on a rotary shaker (MaxQ 2000 Open-Air Platform Shaker; ThermoFisher Scientific, USA) at 240 rpm at room temperature for 40 min and filtered using a cotton cloth. The filtrate was then centrifuged (NF200, year 2009, SN 02-4005, Turkey) at 10000 x g, 4°C for 10 min. The supernatant was used as a crude enzyme.

### 2.7. Assay for Acid Protease Activity

Protease activity was determined using hemoglobin as a substrate [18]. Enzyme preparation (0.5 mL), suitably diluted, was mixed with 1 mL of 2% (w/v) hemoglobin prepared in 100 mM glycine HCl buffer (pH 3.0), and the mixture was incubated in a water bath at 50°C for 10 min. The reaction was terminated by adding 2 mL trichloroacetic acid 5% (w/v). The mixture was allowed to stand at room temperature for 15 min and then centrifuged (NF200, year 2009, SN 02-4005, Turkey) at 10,000 *×* g for 15 min to remove the precipitate. The absorbance of the soluble fraction was measured at 280 nm. A standard curve was generated using tyrosine solutions. One unit of protease activity was defined as the amount of enzyme required to liberate 1 *μ*g of tyrosine per min under the experimental conditions. The tyrosine standard curve and protease activity of the enzyme were calculated.

### 2.8. Tyrosine Standard Curve

The protease activity of the enzyme was determined using the tyrosine standard curve. The absorbance of standard tyrosine solution (tyrosine solutions at 0–250 *μ*g/mL) was measured at 280 nm (*A*280) using a spectrophotometer (UV-VIS, Liantrinsat, and Model-CF728YW-UK). Then, a standard curve was plotted using the OD reading and the tyrosine concentrations (Figure 1). The OD reading of the samples (assay conducted using fungal protease) was converted to the liberated amino acid (tyrosine) using the formula generated from the standard curve. Finally, the tyrosine liberated during the assay was converted to protease activity using the following formula:

(2) $$\begin{array}{c} \text{PA }\left(\frac{U}{mL}\right)=\frac{µ\text{Tyr}*V_{t}}{V_{s}*T*V_{a}}, \end{array}$$

where PA: protease activity, *µ*Try: *µ*g of tyrosine equivalent released, *V*_*t*_: total volume of the assay in mL, *V*_*s*_: sample volume, *T*: reaction time in minutes, and *V*_*a*_: volume of the assay used for absorbance measurement.

### 2.9. Assay for Milk Clotting Activity

Milk clotting activity was determined according to the method of Arima et al. [19], which is based on the visual evaluation of the appearance of the first clotting flakes, and expressed in terms of Soxhlet units (SU). One Soxhlet unit is defined as the amount of enzyme that clots 1 mL of the substrate in 40 min at 35°C. To perform the assay, 0.1 mL of the sample was added to a glass test tube containing 1 mL of reconstituted skim milk solution (10 g skim milk powder dissolved in 100 mL of 0.01 M CaCl_2_ solution) pre-incubated at 35°C for 10 min. The mixture was mixed well, and the clotting time *t* (s) was measured using a chronometer. The clotting activity was calculated using the following formula:

(3) $$\begin{array}{c} \text{SU}=\frac{\left(2400\times 1\times D\right)}{\left(0.1\times t\right)}, \end{array}$$

where *D* is the dilution factor and *t* is the clotting time in seconds.

### 2.10. Morphological Characterization

The cultural characteristics (colony growth rate, colony texture, colony color, colony size, and degree of sporulation) of the fungal isolates were studied by inoculating them on Czapek Dox agar (CDA), potato dextrose agar (PDA), and malt extract agar (MEA) as described by Zulkifli and Zakaria [20]. The microscopic structures of the isolates were observed under the microscope after having prepared them on a slide culture and identified to the genus level based on species descriptions [21, 22].

### 2.11. Optimization of Cultural Conditions and Media Composition for the Production of Acid Protease under SSF

#### 2.11.1. Experimental Setup for Preliminary Screening for Production of Acid Protease in SSF

The experimental setup for solid-state fermentation was according to the study by Fernández-Lahore et al. [16] with slight modification. Two types of fermentation media were used for solid-state fermentation. The 1st media contained 10 g of wheat bran (WB) and 2 g of skim milk powder moistened by 12 mL HCl (0.2 M). The 2nd media contained 10 g of wheat bran (WB) and 2 g of skim milk powder moistened by 10 mL mineral solutions. The flasks were then incubated at 30°C for six days.

#### 2.11.2. Effect of Substrate on Acid Protease Production

Screening of the media composition for acid protease production was performed by a one-variable-at-a-time approach [22, 23]. Thus, 0.5 mL of spore suspension (10^6^ spores/mL) from potential fungal isolates were inoculated into the flask containing 10 grams of each substrate (wheat bran, Rice Bran) in 250 Erlenmeyer flasks moistened by 12 mL HCl (0.2 M)/salt solution and incubated at 30°C for 144 h. The crude enzyme was extracted as previously described and assayed for acid protease activity. After having tested the effect of substrates on enzyme production, the highest enzyme-producing substrate was selected and tested for further optimization.

#### 2.11.3. Effect of Incubation Time

The effect of incubation time on acid protease production was studied by inoculating the flasks containing wheat bran, the best substrate, with 0.5 mL of spore suspension (10^6^ spores/mL) and incubated at 30°C for different periods ranging from 24 h to 144 h [23]. The acid protease activity was determined as indicated in Section 2.7.

#### 2.11.4. Effect of Incubation Temperature

The fungal spores inoculated into the SSF medium in 250 mL Erlenmeyer flasks were incubated at temperatures of 20°C, 25°C, 30°C, 35°C, 40°C, and 45°C for 120 h to determine the optimum temperature. Then, the obtained optimum temperature was used for further study [23].

#### 2.11.5. Effect of Inoculum Size

The effect of inoculum size on acid protease production was studied by inoculating 0.2 mL, 0.5 mL, 1 mL, 1.5 mL, and 2 mL of (3.2 × 10^6^ spores/mL) spore suspension into SSF media. Then, the inoculated flasks were incubated at the optimum temperature for 120 h [23].

#### 2.11.6. Effect of Moisture Content

The effect of initial moisture content for enzyme production was tested by moistening substrate with distilled water at different percentages of moisture content 45%, 50%, 55%, 60%, 65%, and 70% to find out the best percentage for enzyme production [24].

#### 2.11.7. Effect of Initial Media pH

The effect of initial media pH on acid protease production was optimized by adjusting the SSF medium to pH 2.0, 2.5, 3.0, 3.5, 4.0, 4.5, 5.0, 5.5, 6.0, 6.5, and 7.0 using HCl or diluted NaOH [23].

### 2.12. Partial Purification

#### 2.12.1. Acetone Precipitation

The crude enzyme extract was precipitated with chilled acetone (acetone or 75% acetone). Two volumes of chilled acetone were slowly added to the extract, and the precipitate was allowed to settle for 1 h at −18°C to permit complete precipitation. The precipitated proteins were then separated by centrifuging at 10000 × g and 4°C for 10 min. The pellet was dried in the open air for 30 min to remove trace amounts of acetone and dissolved in 0.02 M phosphate buffer, pH 6.0 [26].

#### 2.12.2. Ammonium Sulfate Precipitation

The crude enzyme extract was precipitated with ammonium sulfate according to the study by Nouani et al. [26]. Accordingly, 300 mL of the crude extract was added to 1200 mL saturated ammonium sulfate (100%) to precipitate. Then, the enzymatic solution was decanted for one night at 4°C and centrifuged at 10000 × g for 10 min at 4°C, and the pellet was suspended in the phosphate buffer (0.02 M; pH 6).

### 2.13. Enzyme Characterization

#### 2.13.1. Determination of Optimum pH and Its Stability

The optimum pH of the enzyme preparation was studied over a pH range of 2.0–7.0 at 50^o^C using 2% (w/v) hemoglobin as described previously. For studying pH stability, the crude enzyme was incubated in buffers of different pH values in the range of pH 3.0–7.0 for 1 h at 4°C. Residual proteolytic activity was then determined under standard assay conditions. The following buffer systems were used: 100 mM glycine-HCl buffer for pH 3.0 and 3.5, 100 mM sodium acetate buffer for pH 4.0–6.0, and 100 mM potassium phosphate buffer for pH 6.5 and 7.0 [22].

#### 2.13.2. Determination of Optimum Temperature and Its Stability

To investigate the effect of temperature, proteolytic activity was tested at different temperatures (20, 25, 30, 35, 40, 45, 50, 55, 60, and 65°C) using hemoglobin as a substrate for 5 min at pH 5.0. Thermal stability was examined by incubating the enzyme at 30, 40, 50, 60, and 70°C for 60 min. After 60 min, the remaining enzyme activity was determined at optimum pH and temperature. The non-heated enzyme was considered 100% [22].

### 2.14. Data Analysis

Data analyses were performed using SPSS software, version 23 (Inc. Cary NC USA). All experiments were carried out in triplicate. Analysis of variance (ANOVA) and means comparisons were done by SPSS and SigmaPlot, version 12.

## 3. Results

### 3.1. Primary Screening of Protease-Producing Fungi

A total of thirty-two (32) fungal isolates were isolated from the soil samples, of which 12 isolates (38%) showed clear zone hydrolysis on skim milk agar media up on primary screening. The fungal isolates were shown significant clear zone diameter and relative enzyme activity (REA) ranging from 6.7 mm and 21.3 mm and between 0.4 and 0.74, respectively (Table 1). Among the isolates, the highest REA was recorded by the isolates H1W1 (0.8) and Z1BL1 (0.74) (Table 1). The spore concentration of all the 12 isolates was determined using the Neubauer chamber and was in the range between 1.2 × 10^6^ and 4.4 × 10^8^ spores/mL (Table 1).

### 3.2. Secondary Screening of Acid Protease under Solid-State Fermentation (SSF)

All the twelve isolates that showed a relative protease activity ≥0.4 were subjected for further screening in solid-state fermentation. Out of the twelve isolates, 9 isolates showed an acid protease activity under SSF (Table 2). The highest protease activity (78.2 U/mL) was recorded from the isolate Z1BL1, whereas the lowest protease activity (31.7 U/mL) was noticed from the isolate Z1Y2 under SSF. Hence, the isolate Z1BL1 that showed the highest acid protease activity was selected for further study.

### 3.3. Morphological Characterization of Potential Fungal Isolates

**Figure 2:**
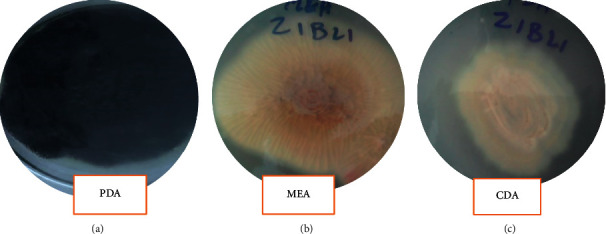
Macroscopic features of the isolate Z1BL1.

**Figure 3:**
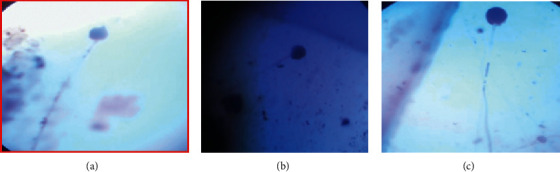
Microscopic structures of the isolate Z1BL1.

The potential fungal isolate Z1BL1 was identified and categorized under the genus *Aspergillus* based on microscopic and macroscopic characteristics (Table 3, Figures 2 and 3).

### 3.4. Optimization of Media Composition and Growth Conditions for Acid Protease Production by the Isolate Z1BL1

#### 3.4.1. Effect of Substrate Types for Acid Protease Production

**Figure 4:**
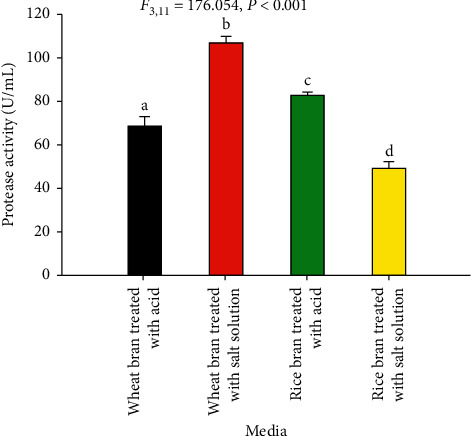
Screening of two agro-industrial residues for acid protease production from the filamentous fungal isolate Z1BL1. Mean is the average of three measurements. Different letters (a, b, c, d) designate significantly different means as determined by the SPSS test (*P* < 0.001); temperature 30°C; initial media pH 4.5.

In the present study, two solid substrates (wheat bran and rice bran) moistened by acid/mineral salt solution were used for acid protease production. All the substrates treated by acid and mineral solutions produced significant protease activity. However, the maximum protease activity (107.4 U/mL) was obtained from the potential isolates (Z1BL1) using wheat bran treated with mineral solution, whereas the least protease activity (48.5 U/mL) was recorded using rice bran treated with the same mineral solution (Figure 4).

#### 3.4.2. Effect of Incubation Period

The effect of incubation time on the production of acid protease is shown in Table 4. The highest protease activity (95.2 U/mL) was recorded at 120 h. Further increase in the incubation time reduced the enzyme activity.

#### 3.4.3. Effect of Incubation Temperature

**Figure 5:**
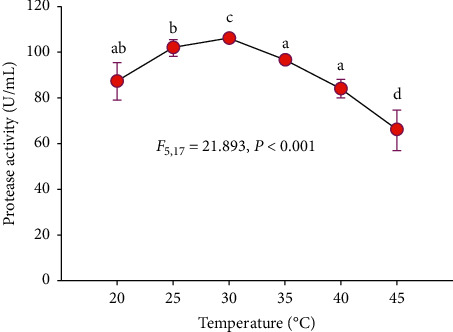
Effect of incubation temperature on acid protease production from the filamentous fungal isolate Z1BL1 in SSF. Different letters (a, b, c, d) designate significantly different means as determined by the SPSS test (*P* < 0.001); temperature 30°C; initial media pH 4.5.

In the present study, the highest protease activity (106.1 U/mL) was obtained when SSF carried out at a temperature of 30°C, while the lowest protease activity was achieved at 45°C (Figure 5).

#### 3.4.4. Effect of Inoculum Size

**Figure 6:**
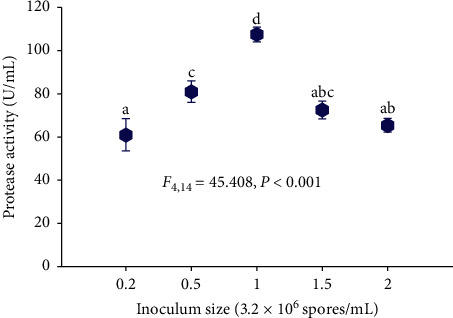
Effect of inoculum size on the production acid protease by the isolate Z1BL1. Different letters (a, b, c, d) designate significantly different means as determined by the SPSS test (*P* < 0.001); initial media pH 4.5; temperature 30°C.

The effect of inoculum size on acid protease production by the isolate Z1BL1 is shown in Figure 6. The maximum protease activity was obtained at an inoculum size of 1 mL (3.2 × 10^6^ spores/mL), whereas the lowest activity was recorded at 0.2 mL (3.2 × 10^5^ spores/mL).

#### 3.4.5. Effect of Media Moisture Content

**Figure 7:**
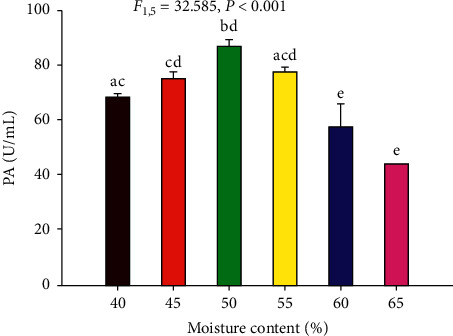
Effect of moisture content on acid protease production by the filamentous fungal isolate Z1BL1 in SSF. PA: protease activity. Different letters (a, b, c, d, e) designate significantly different means as determined by the SPSS test (*P* < 0.001); wheat bran moistened with the salt solution; initial media pH 5; temperature 30°C.

The effect of moisture content on acid protease production is shown in Figure 7. Accordingly, the maximum enzyme production (87.4 U/mL) was obtained at 50% moisture content, whereas the lowest (44.7 U/mL) was recorded at a moisture content of 65%.

#### 3.4.6. Effect of Initial Media pH

**Figure 8:**
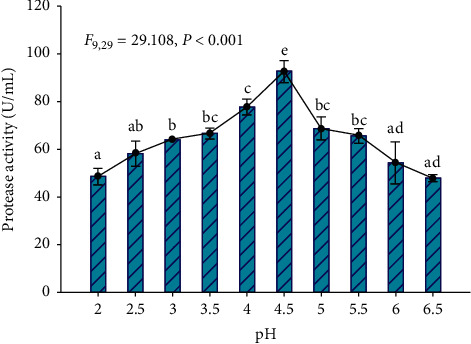
Effect of initial media pH on the production of acid protease from filamentous fungi Z1BL1 in SSF. Different letters (a, b, c, d, e) designate significantly different means as determined by SPSS test (*P* < 0.001); wheat bran moistened with a salt solution; temperature 30°C.

Maximum acid protease production (92.6 U/mL) by the isolate Z1BL1 was recorded at initial media pH 4.5. The protease activity was sharply decreased on both sides of pH 4.5 (Figure 8).

### 3.5. Enzyme Characterization

The crude enzyme was partially purified by acetone and ammonium sulfate precipitation, and the partially purified enzyme was used for characterization. Purification of the enzyme by acetone (NH_4_)_2_SO_4_ increased the ratio of MCA/PA while reducing the MCA and PA (Table 5). The highest ratio was recorded from the acetone-precipitated enzyme. Thus, based on the protease activity and ratio (MCA/PA) recorded from the enzymes, the acetone-precipitated enzyme was subjected to further enzyme characterization.

#### 3.5.1. Effect of Temperature on Enzyme Activity and Stability

**Figure 9:**
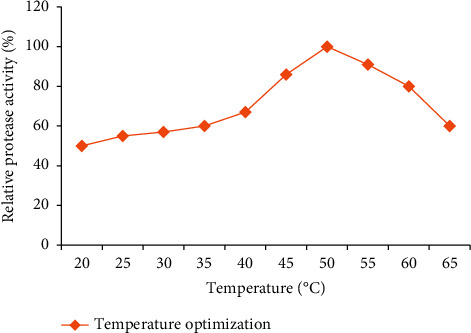
Temperature optimization of PA for acetone-precipitated enzyme from the isolate Z1BL1.

**Figure 10:**
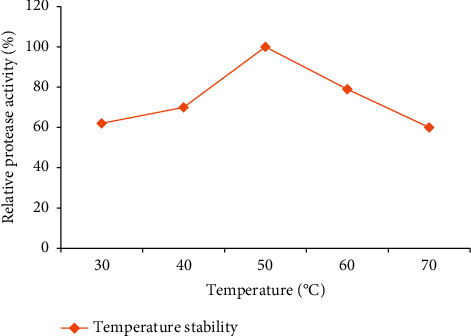
Temperature stability of PA for acetone-precipitated enzyme from the isolate Z1BL1.

As shown in Figure 9, the crude enzyme precipitated with chilled acetone showed significant protease activity at a temperature range from 40°C to 60°C. However, the highest activity was recorded at a temperature of 50°C. The enzyme activity gradually increased with increasing temperature, followed by a steep decrease at temperatures above 50°C (Figure 9). The enzyme retained stable (70–79% of its activity) upon its exposure to a temperature between 40°C and 60°C (Figure 10) for 1 h.

#### 3.5.2. Effect of pH on Enzyme Activity and Stability

**Figure 11:**
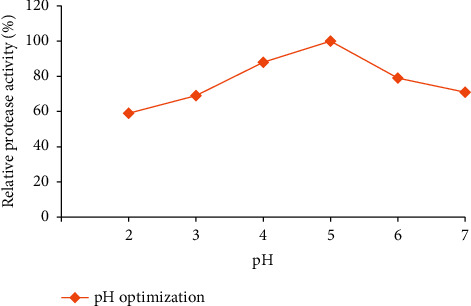
pH optimization of PA for acetone-precipitated enzyme from the isolate Z1BL1.

In the present study, the optimum pH for the activity of acetone-precipitated enzyme (97.1 U/mL) was obtained at pH 5.0 (Figure 11).

**Figure 12:**
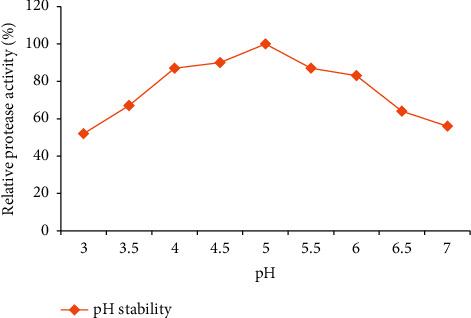
pH stability test for acetone-precipitated enzyme from the isolate Z1BL1.

The pH stability test for acetone-precipitated enzyme from the isolate Z1BL1 was stable from pH 4–6 as shown in Figure 12. The enzyme retained significant activity (83–90%) upon its exposure at a pH ranging from 4 to 6.

## 4. Discussion

In the current study, 12 (38%) fungal isolates showed a clear zone of hydrolysis on skim milk agar media during primary screening. Skim milk agar plate assay allows qualitative determinations of protease activity, and the hydrolysis zone produced on the agar could be related to the amount of protease produced by the fungus [13, 28, 29]. The fungal isolates produced a clear zone diameter and relative enzyme activity between 6.7 mm and 21.3 mm and 0.40 and 0.80 in 48 hours, respectively. Similar to the present study, substantial clear zone hydrolysis was reported by *Mucor* sp. [30] and *Aspergillus niger* FFB1 [24] on skim milk agar and agar media, respectively, whereas the clear zone diameter (8 mm–19.25 mm) and relative enzyme activity (1.07–2.09) recorded from various filamentous fungi using skim milk agar media up to 48 hours were higher than this study [14]. The variation in clear zone diameter and relative enzyme activity could be attributed to the low media pH used in this study.

Out of the 12 isolates subjected to secondary screening, 9 isolates were shown significant protease activity (between 31.7 U/mL and 78.2 U/mL) under SSF. This could be due to those filamentous fungi preferred SSF for the production of enzymes [23]. Similar protease activities were also recorded by various *Aspergillus* spp. under SSF [24, 31–33].

The potential fungal isolate Z1BL1 that exhibited the highest protease activity under SSF was identified and categorized under genus *Aspergillus* based on macroscopic and microscopic characteristics (Table 3, Figures 2 and 3). In comparison with the present study, filamentous fungal isolates collected from the Petaling Jaya region (Malaysia) were identified and classified into different species under the genus *Aspergillus* [14]. In another study, Afzal et al. [34] reported the identification of filamentous fungi into the genus *Aspergillus* based on the morphological features.

Moisture content is one of the significant factors in enzyme production in solid-state fermentation [35] since microbial growth and product formation occur at or near the surface particle with an optimized water level that controls the water activity (*a*_*W*_) [15]. In this study, the highest protease activity was observed from the crude enzyme produced at 50% moisture content (Figure 7). Similar to the present study, the maximum milk-clotting enzyme from *Aspergillus* spp. was produced at 55% moisture content under SSF [7, 31]. The high moisture content of the fermentation media decreased the porosity and oxygen transfer that may affect the enzyme production [36].

The optimum initial media pH for acid protease production by the isolate Z1BL1 was found at pH 4.5 (Figure 8). Further increasing the media pH significantly reduced enzyme production, and this implies that the fungus (Z1BL1) prefer an acidic media pH for inducing acid protease. A similar observation was reported by Yegin et al. [37] on the production of aspartic protease from *Mucor mucedo.* In other studies, maximum acid protease activity from *Rhizopus oligosporus* HIS13 [38] and *Aspergillus* species [33] under SSF was also obtained at initial media pH 5. Generally, changes in pH during the SSF procedure were rarely controlled, except the initial pH of the substrate that is adjusted before inoculation [24].

The protease activity was measured for 144 h within 24 h interval, and the maximum yield (95.2 U/mL) was noticed at 120 h of the incubation period (Table 4). Further incubation significantly reduced the enzyme production. The reduction in enzyme yield after the optimum incubation period was probably due to the depletion of nutrients available for microbial growth [15]. The maximum enzyme activity recorded from *M. circinelloides*, *A. oryzae* MTCC 5341, and *Aspergillus* spp. on 120-h fermentation time under SSF was comparable with the present study [15, 39, 40].

In this study, acid protease production carried out at 30°C was the most suitable incubation temperature for the isolate Z1BL1 with protease activity of 106.1 U/mL (Figure 5), and any fluctuations from this optimum temperature significantly reduced the enzyme activity. The production of the enzyme is directly related to the biomass of the fungi, which implies optimum growth temperature for the isolate Z_1_BL_1_ was at 30°C. Similar to the current study, optimum acid protease production by *Aspergillus* spp. was obtained at 30°C [24, 32, 33, 39]. In another study, Shieh et al. [41] also reported maximum milk-clotting proteases production by *Amylomyces rouxii, Mucor pusillus,* and *Mucor* J20 at 30°C. A maximum acid protease activity was also recorded from *A. oryzae* HG76 at 30°C and showed a significant decrease when the temperature goes higher or lower [42].

The selection of the best substrate for enzyme production in SSF process is governed by several factors, mainly related to the cost and availability of the substrate material, and thus may involve screening of agricultural waste products [43]. In the present study, the suitability of two substrates (wheat bran and rice bran) moistened with acid/mineral solution was verified for protease production in SSF. The highest enzyme activity was obtained from wheat bran moistened with mineral solution. The variation in protease activity between the solid substrates could be due to differences in the biochemical composition and in particle size, which is partly related to porosity even though the particle size of each substrate was not determined [44]. In other studies, wheat bran is considered the best substrate for the production of acid protease from *A. oryzae* MTCC 5341 [39], other *Aspergillus* species [24, 42], and *Mucor* species [45].

The size of the inoculum is an important biological factor that determines biomass production during fermentation. A highly concentrated inoculum may produce excessive biomass leading to the rapid depletion of nutrients needed for the rapid growth of the culture and production of metabolites, while a lower inoculum density may give insufficient biomass inducing low yields of products [24, 46, 47]. Thus, determining an appropriate inoculum size in SSF is vital for optimum biomass growth and hence maximum enzyme production. In the present study, the highest protease activity (107.4 U/mL) of the crude enzyme produced from Z1BL1 was obtained using an inoculum size of 1 mL (3.2 × 10^6^ spores/mL) (Figure 6). Differently, maximum protease production from *A. oryzae* NRRL 1808 [46] and *Mucor circinelloides* [15] was reported at 8** × **10^8^ spores/mL and 10^8^ spores/mL, respectively.

The extra addition of inoculum size to 6.4** × **10^6^ spores/mL substantially decreased acid protease activity. The decrease in enzyme activity that has been observed with higher inoculum size could be due to the shortage of the nutrients available for the larger biomass and faster growth of the culture. Hence, a balance between the proliferating biomass and available material is vital for maximum enzyme production [46].

Characterization of protease is an important practice to determine the optimum temperature and pH of the enzyme for its application. In this study, the crude enzyme precipitated using acetone was used for characterization. The partially purified enzyme showed maximum activity at temperature 50**°**C and stability at a temperature between 40°C and 60°C. The thermostability of the enzyme showed in this study corresponds with the potential applications of the enzyme in food industries such as bakery and brewery [4, 33], and the highest activity of acid protease from *Aspergillus oryzae* MTCC 5341 at 55°C and its stability between 40 and 57°C were also reported [30]. The acid protease was most active at pH 5.0, and the protease activity decreased significantly below and above this value. The enzyme was active from pH 4 up to 6 as shown in Figure 12. This suggests that the enzyme is active at acidic pH and appropriate for the food industry and beverage industry [5]. Similarly, Oseni [48] reported the highest activity of acid protease from *Aspergillus* spp. at pH 5. Optimum pH values between 3.0 and 5.5 have been reported for protease activities of other fungi, such as *Penicillium camemberti* (pH 3.5) [49], and *Rhizopus oryzae* (pH 5.5) [50]. The optimum pH of NRRL 1785 protease, which exhibited an optimum level at pH 4.0, was comparable with the current study [51]. Thus, the stability of the enzyme in the present study corresponds with the pH used for food processing for the food and beverage industries [33].

## 5. Conclusions

Filamentous fungi isolated from dairy and grape farm soil have the potential to produce acid protease. From primary and secondary screening, isolate Z1BL1 was selected as a potential fungus for acid protease production under SSF. The potential isolate was successfully identified and categorized under genus *Aspergillus* using macroscopic and microscopic features. Optimization of media composition and growth conditions of the isolate Z1BL1 slightly increased the acid protease production. Characterization of the partially purified enzyme confirms that the enzyme works best at pH 5 and 50**°**C. Thus, based on the above findings, isolate Z1BL1 could be a potential candidate for production of acid protease applicable in the food industry.

## Figures and Tables

**Table 1:** Clear zone diameter of fungal isolates on skim milk agar media.

No.	Isolates	Spore concentration/mL	CD (mm), mean ± SEM	CZD (mm), mean ± SEM	REA
1	Z_1_BL_1_	6.54 × 10^7^	28.7 ± 1.5^b^	21.3 ± 1.5^a^	0.74
2	H_1_BL_1_	3.60 × 10^6^	14.7 ± 0.8^a^	9.0 ± 0.6^cd^	0.61
3	Z_1_BL_3_	2.64 × 10^8^	19.0 ± 1.5^a^	11.7 ± 1.2^c^	0,61
4	SW_2_W_1_	1.81 × 10^7^	19.0 ± 2.0^a^	12.3 ± 0.3^c^	0.65
5	SW_1_BBr	8.12 × 10^7^	14.3 ± 1.5^a^	6.7 ± 1.5^d^	0.46
6	SW_1_YW_1_^+^	1.4 × 10^6^	15.7 ± 1.2^a^	8.3 ± 0.3^cd^	0.52
7	Code 3	2.6 × 10^6^	15.7 ± 0.3^a^	10.0 ± 0.6^cd^	0.63
8	Z_1_Y_2_	1.5 × 10^6^	17.3 ± 0.8^a^	7.0 ± 0.5^d^	0.40
9	H_1_W_1_	1.2 × 10^6^	20.3 ± 6^a^	16.3 ± 1.2^b^	0.80
10	SW_1_WY_1_	2.2 × 10^7^	18.3 ± 2.4^a^	11.7 ± 0.3^c^	0.63
11	Z_1_BL_4_	4.4 × 10^8^	16.7 ± 0.6^a^	10.0 ± 0.6^cd^	0.60
12	H_1_Gr_1_	2.6 × 10^6^	15.3 ± 1.5^a^	9.3 ± 0.3^cd^	0.60

Mean is the average of three measurements. SEM: standard error of the mean; REA: relative enzyme activity; CD: colony diameter; CZD: clear zone hydrolysis diameter. Different letters (a, b, c, d) designate significantly different means as determined by the SPSS test (*P* < 0.05).

**Table 2:** Acid protease activity of fungal isolates under SSF.

No.	Isolates	Adjusted spore concentration/mL	PA (U/mL), mean ± SEM
1	Z_1_BL_4_	3.6 × 10^6^	54.9 ± 1.7^a^
2	H_1_Gr_1_	1.3 × 10^6^	38.8 ± 2.2^b^
3	Z_1_BL_1_	3.2 × 10^6^	78.2 ± 1.5^a^
4	Z_1_BL_3_	3.1 × 10^6^	49.2 ± 1.7^ab^
5	SW_2_W_1_	2.2 × 10^6^	70.1 ± 2.8^a^
6	H_1_BL_1_	1.8 × 10^6^	46.6 ± 4.0^b^
7	Z_1_Y_2_	1.5 × 10^6^	31.7 ± 1.7^b^
8	H_1_W_1_	1.2 × 10^6^	47.2 ± 2.8^ab^
9	SW_1_WY_1_	2.0 × 10^6^	51.7 ± 1.7^ab^

Mean is the average of three measurements. SEM: standard error of the mean; PA: protease activity. Different letters (a, b) designate significantly different means as determined by the SPSS test (*P* < 0.05).

**Table 3:** Macroscopic characteristics of potential fungal isolates.

Isolates	Media	Colony growth	Colony texture	CD, mean ± SEM	Obverse colony color	Reverse colony color	Degree of sporulation
Z1BL1	MEA	Medium	Floccose (cottony)	28.3 ± 1.5	Black	Dark black	High
	CDA	Low	Floccose (cottony)	21 ± .5	Dark white to gray	Pink	High
	PDA	High	Floccose (cottony)	40.3 ± 1.2	Dark black	Blue-black	High

H_1_Gr_1_	MEA	Medium	Rough	30.7 ± 2.2	Green	White gray	Low
	CDA	Medium	Rough	29.6 ± .8	White gray	Green	Low
	PDA	High	Rough	43.0 ± 1	Green	White to gray	Medium

H_1_W_1_	MEA	Medium	Floccose (cottony)	31.3 ± 2	White to brown	Brown	Low
	CDA	Low	Floccose (cottony)	24.5 ± .6	White to brown	Dark brown	Low
	PDA	High	Floccose (cottony)	38.1 ± 1.1	Yellow	White to yellow	Low

SW_2_W_1_	MEA	High	Floccose (cottony)	40.4 ± 3	Blue	Brown	Medium
	CDA	Low	Floccose (cottony)	23.9 ± 5	Brown	White to brown	Medium
	PDA	High	Floccose (cottony)	45.0 ± .5	Brown	Brown with white	High

SW_1_WY_1_	MEA	Medium	Rough	25.5 ± 2.3	White to yellow	White and brown	Medium
	CDA	Medium	Rough	33.5 ± 1	Yellow	Yellow at white	Medium
	PDA	High	Rough	38.7 ± 1.2	White yellow	Light yellow	High

Z_1_BL_3_	MEA	High	Floccose (cottony)	41.6 ± 1.4	Blue-black	Black	High
	CDA	Medium	Floccose (cottony)	30.0 ± 2	Black	Dark black	Medium
	PDA	High	Floccose (cottony)	43.0 ± 1.5	Dark black	Blue-black	High

Z_1_Y_2_	MEA	Medium	Rough	29.4 ± .5	Yellow	White	Low
	CDA	Very low	Rough	13.7 ± 2.3	Yellow	Brown	Low
	PDA	Medium	Rough	31.5 ± 1.5	White	White	Low

H_1_BL_1_	MEA	Low	Floccose (cottony)	20.1 ± 1.9	Blue-black	Dark black	Low
	CDA	High	Floccose (cottony)	36.6 ± 1.4	Black	Black	Low
	PDA	High	Floccose (cottony)	40.3 ± .8	Dark black	Blue-black	High

Z_1_BL_4_	MEA	High	Floccose (cottony)	36.0 ± 2	Blue-black	Black	Medium
	CDA	Low	Floccose (cottony)	21.0 ± 1	Black	Dark black	Low
	PDA	High	Floccose (cottony)	36.2 ± 1.7	Black	Blue-black	High

CD: colony diameter; MEA: malt extract agar; CDA: Czapek Dox agar; PDA: potato dextrose agar. Colony growth—Very low: <15 mm; Low: 15–25 mm; Medium: 25–35 mm, High: 35–45 mm; Very high: >45 mm [27].

**Table 4:** Effect of incubation period on acid protease production by the isolate Z1LB1.

No.	Incubation period (in hour)	PA (U/mL) mean ± SEM
1	24	—
2	48	36.8 ± 5^a^
3	72	56.3 ± 7^b^
4	96	88.0 ± 5.2^c^
5	120	95.2 ± 7^c^
6	144	71.8 ± 4.9^bc^

PA: protease activity; SEM: standard error of the mean. Mean is the average of three measurements. Different letters (a, b, c) designate significantly different means as determined by the SPSS test (*P* < 0.001); temp. 30°C; initial media pH 4.5.

**Table 5:** Protease activity of the crude enzyme and partially purified enzyme from the isolate Z1BL1.

Sample	MCA (U/mL), mean ± SEM	PA (U/mL), mean ± SEM	Ratio MCA/PA
Crude enzyme	99.2 ± 1.7	107.6 ± 1	0.92
Acetone-precipitated enzyme	75.7 ± 1.0	50.4 ± 1.1	1.50
(NH_4_)_2_SO_4_ precipitated enzyme	64.7 ± 2.3	48.2 ± 4.0	1.34

MCA: milk clotting activity; PA: protease activity; ratio: the ratio of MCA/PA; SEM: standard error of the mean; mean: an average of three measurements.

## Data Availability

The experimental data used to support the findings of this study are included within the article.
